# Advanced Mucosal Melanoma Therapies: Current Status and Future Directions

**DOI:** 10.1007/s11864-025-01372-y

**Published:** 2026-04-07

**Authors:** Yiqun Zhang, Dawei Zhao, Di Wu

**Affiliations:** 1 Department of Oncology, Cancer Center, The First Hospital of Jilin University, 1 Xinmin St, Changchun, 130021 China; 2 Department of Breast Tumor, Jilin Cancer Hospital, No.1066 Jinhu Road, Changchun, 130012 China

**Keywords:** Mucosal melanoma, Targeted therapy, Immunotherapy, Combination therapy

## Abstract

Mucosal melanoma (MM) represents a rare malignancy in Caucasian populations but constitutes one of the predominant melanoma subtypes among non-Caucasian ethnic groups. Due to its anatomic occult nature, a significant proportion of patients with MM present with advanced-stage disease at diagnosis. Characterized by distinct genomic profiles and an immunosuppressive tumor microenvironment, MM exhibits suboptimal responses to current targeted drugs and immune checkpoint inhibitors (ICIs). Combination immunotherapy can overcome immune evasion by enhancing the infiltration of tumor-specific antigen-reactive T lymphocytes, thereby exhibiting potentiated anti-tumor activity. However, the rarity of MM has posed significant barriers to better understanding immunotherapy resistance mechanisms and investigating novel therapies. Consequently, there is a critical unmet need for establishing standardized treatment guidelines to improve survival outcomes in advanced MM. This article comprehensively reviews current status in the treatment of advanced MM. It also discusses potential future directions for treatment based on recent advances from clinical trials and basic research.

**Supplementary Information:**

The online version contains supplementary material available at 10.1007/s11864-025-01372-y.

## Introduction

**Fig. 1:**
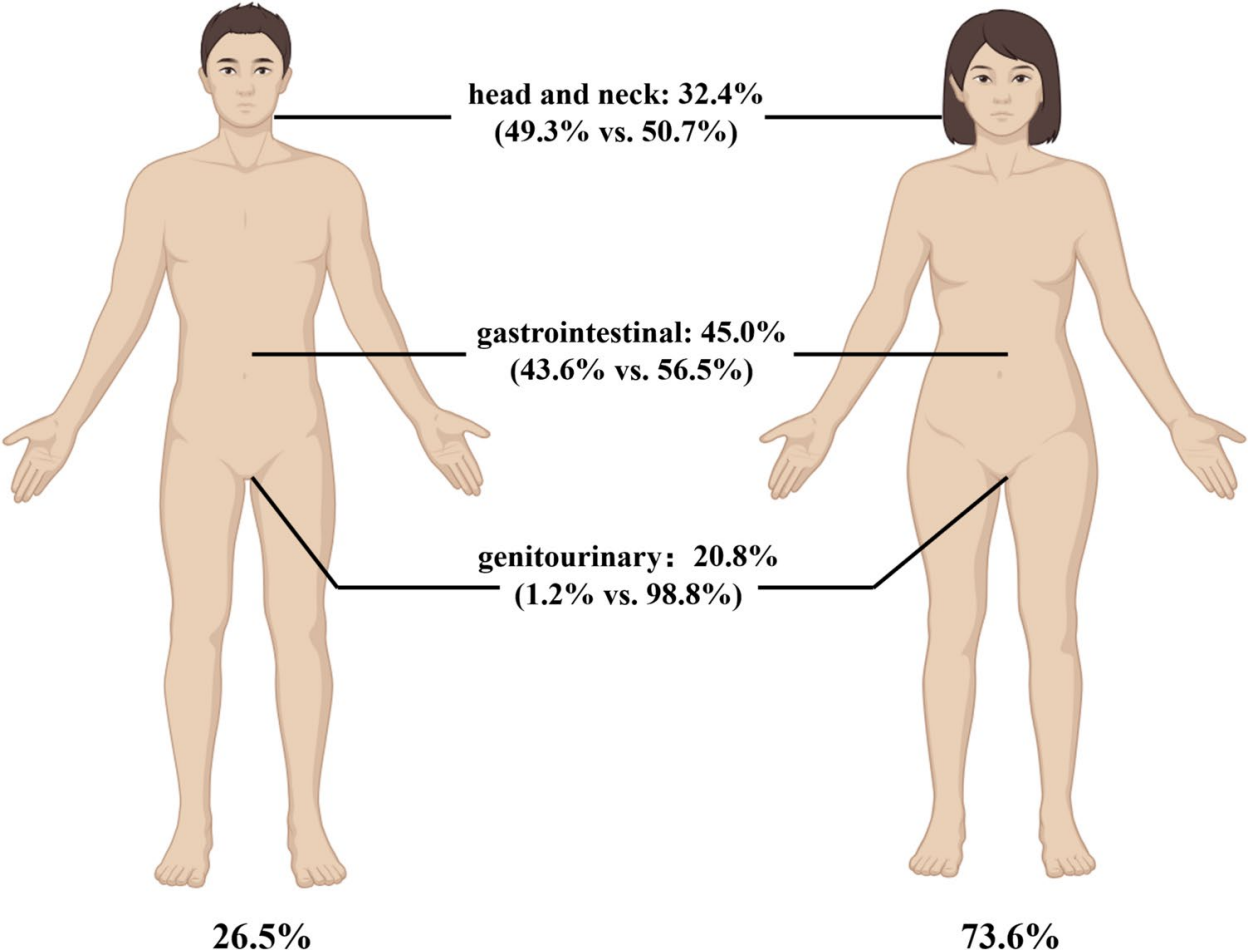
Distribution of MM anatomical and gender location.

Mucosal melanoma (MM) originates from melanocytes in the mucosa of the head and neck (nasopharynx and oral cavity), digestive tract, female genital tract, urinary tract, and respiratory system. The most common primary site of MM is the gastrointestinal (45.0%), followed by the head and neck region (32.4%) and genitourinary (20.8%) [1] (see Fig. 1). The occult anatomical locations of MM present a significant diagnostic challenge, frequently leading to delayed diagnosis. Among Caucasians, MM comprises only 1.3% of all melanomas [1], whereas among Asians, it represents 9.5%−30.5% of all melanomas [2–5], ranking second only to acral melanoma. The rarity of MM hampers progress in both basic and clinical research.

With the application of immune checkpoint inhibitors (ICIs) and targeted therapies, the prognosis for patients with advanced cutaneous melanoma (CM) has been greatly improved, elevating the 5-year OS rate from less than 10% to 40%−50% [6]. In contrast, MM is one of the most aggressive subtypes of melanoma, with a 5-year OS rate of only 14% in advanced cases [7].The tumor microenvironment (TME) of MM is characterized by an immunosuppressive state, which contributes to its poor response to ICIs. This immunosuppression is attributed to a combination of factors, including a low tumor mutational burden [8, 9], reduced programmed death ligand 1 expression [10–12], and diminished infiltration of tumor-infiltrating lymphocytes [13]. Furthermore, the incidence of BRAF mutations is significantly lower in MM compared to CM, substantially restricting the clinical utility of BRAF inhibitors in the treatment of advanced MM [8].

This article provides a comprehensive review of the current treatment landscape for advanced MM and proposes potential innovative therapeutic strategies based on recent advances in clinical and basic research.

## Current Status

### Chemotherapy

For metastatic melanoma, dacarbazine has been one of the most commonly used chemotherapy drugs [6–9]. Multiple retrospective analyses have demonstrated that first-line biochemotherapy incorporating dacarbazine confers objective response rates (ORR) ranging from 36 to 47% in patients with advanced MM. These regimens are associated with a median progression-free survival (PFS) of 3 to 6.2 months and a median overall survival (OS) of 9.6 to 12.9 months [14–16]. Despite the need for more effective therapeutic strategies, dacarbazine-based biochemotherapy remains a viable option.

Following the failure of dacarbazine-based chemotherapy, salvage chemotherapy options for patients with metastatic MM are limited. A retrospective study evaluated the efficacy of paclitaxel plus cisplatin (PC) in treating metastatic melanoma. Among 22 patients with non-cutaneous melanoma (including 10 patients with MM), the ORR was 22.7%, with a median PFS of 3.67 months and a median OS of 5.2 months, whereas the median PFS for patients with MM was 1.37 months [17]. Although the efficacy is limited, PC salvage chemotherapy may be a reasonable therapeutic option for heavily pretreated patients with metastatic MM.

#### Immune Checkpoint Inhibitors (ICIs)

Ipilimumab, a cytotoxic T lymphocyte antigen 4 (CTLA-4) inhibitor, as the first ICI shown to prolong the survival of patients with melanoma, is suboptimal for metastatic MM. The ORR for first-line treatment of metastatic MM was 8.2%, with a median PFS of 3 months and a median OS of 12 months [18]. In second-line treatment, the ORR ranged from 6.7% to 12.0%, with a median PFS of 4.3 months and a median OS of 6.4 months [18–21]. Grade 3/4 treatment-related adverse events (TRAEs) occurred in 8% to 12.5% of patients [18, 19, 21].

Compared with ipilimumab, programmed cell death 1 (PD-1) inhibitors such as nivolumab, pembrolizumab, and toripalimab have demonstrated improved clinical efficacy in patients with advanced MM. For patients receiving PD-1 inhibitor monotherapy as first-line treatment, the ORR ranged from 20.0% to 26.0%, with a median PFS of 5.9 to 6.2 months and a median OS of 15.9 to 20.4 months [22–24]. Grade 3 or higher TRAEs were observed in 17% to 20% of patients [22, 24]. In patients receiving subsequent-line treatment, the ORR ranged from 13.3% to 19.0%, with a median PFS of 2.6 to 2.8 months and a median OS of 7.4 to 11.5 months [25–28]. Grade 3/4 TRAEs occurred in 10.0% to 20.6% of patients [26, 27]. However, ICIs have failed to demonstrate a significant survival advantage over conventional chemotherapy in advanced MM.

### Molecular Targeted Therapy

#### BRAF Inhibitors

The frequency of BRAF mutation in MM is relatively low, ranging from 6 to 17% [29–33], thereby limiting the application of BRAF inhibitors in patients with MM. Commonly used BRAF inhibitors include vemurafenib, dabrafenib, and encorafenib. To date, there have been no published clinical trials investigating the efficacy of BRAF inhibitors in the treatment of advanced MM. Only one retrospective study has reported on the outcomes of patients with advanced MM with BRAF V600E mutation treated with vemurafenib, demonstrating an ORR of 20% and a disease control rate (DCR) of 70%. The median PFS was 4.4 months, and the median OS was 8.2 months [34].

#### KIT Inhibitors

The frequency of KIT mutations and amplifications in MM ranges from 15 to 39% [31, 35], with the frequency of KIT mutations approximately ranging from 7% to 19.1% [33, 36, 37]. Currently, only imatinib and nilotinib demonstrate efficacy in advanced MM.

A phase Ⅱ trial enrolled 17 patients with metastatic MM with KIT mutation or amplification. All patients received imatinib treatment, resulting in an ORR of 41%. Interestingly, the ORR among patients with KIT mutations was 64%, while none of the patients with KIT amplification alone achieved clinical remission [38]. For advanced MM with KIT mutations that progress after imatinib treatment, nilotinib may be considered as an alternative. Nilotinib achieved an ORR of 20% in patients with advanced MM who were refractory to a prior KIT inhibitor [39].

#### CDK4 Inhibitors

In MM, the frequency of CDK4 mutation and amplification exceeds 50% [31, 40]. An exploratory clinical study evaluated the efficacy of the CDK4 inhibitor dalpiciclib in patients with advanced head and neck MM harboring CDK4 amplification. The ORR was 6.3%, and the DCR was 81.3%. The estimated median PFS was 9.9 months, and the median OS was not reached [41]. This shows that CDK4 inhibitors and its combination strategy for MM are worth further exploration.

#### Targeted Therapy for MM with NRAS mutation

The frequency of NRAS mutations in MM ranges from 8 to 18% [34–36, 41]. At present, there are no drugs specifically targeting NRAS mutations. Treatment strategies for patients with melanoma with NRAS mutations primarily involve the use of MEK inhibitors to target key signal transduction pathways within the MAPK pathway [42]. Binimetinib represents the first MEK inhibitor to demonstrate activity in NRAS-mutated melanoma. however, its efficacy is suboptimal [43, 44]. A phase II clinical trial evaluating the efficacy and safety of tunlametinib in patients with advanced NRAS-mutant melanoma reported an ORR of 25% and a median PFS of 4.2 months in the MM subgroup [45].

### Combined Treatment

Given the limited efficacy of single chemotherapy, immunotherapy, and targeted therapy in advanced MM, combination therapies have garnered increasing interest, with numerous clinical trials currently ongoing (see Table 1).

**Table 1:** Current clinical trials tailored for patients with unresectable or metastatic mucosal melanoma by combined treatment

Subtype	Agent(s)	NCT number	Sample size	Primary endpoint (s)	Type of study
Mucosal, acral	Nilotinib + Ipilimumab	NCT02978443	*n* = 14	ORR	Phase 2, single-arm
Mucosal	Apatinib + Camrelizumab	NCT03986515	*n* = 40	ORR	Phase 2, single-arm
Mucosal	Axitinib + Nivolumab + Ipilimumab	NCT05384496	*n* = 20	BOR	Phase 2, single-arm
Mucosal	YH003 + Pembrolizumab + Albumin-paclitaxel	NCT05420324	*n* = 43	ORR	Phase 2, single-arm
Mucosal	Toripalimab + CM082	NCT03602547	*n* = 40	ORR	Phase 2, single-arm
Mucosal	Toripalimab + Chemotherapy + Endostar	NCT04472806	*n* = 31	PFS	Phase 2, single-arm
Mucosal, acral	Pembrolizumab + Vactosertib	NCT05436990	*n* = 30	ORR	Phase 2, single-arm
Mucosal	Envafolimab + Chemotherapy + Endostar	NCT06041724	*n* = 46	PFS	Phase 2, single-arm
Mucosal	Toripalimab + Axitinib vs. Toripalimab vs. Axitinib	NCT03941795	*n* = 99	PFS	Phase 2, randomized
Mucosal	Bevacizumab + Paclitaxel + Carboplatin vs. Paclitaxel + Carboplatin	NCT02023710	*n* = 182	PFS	Phase 2, randomized
Mucosal	Nivolumab + DEC-C	NCT05089370	*n* = 30	AEs, RP2D	Phase 1/2, single-arm
Mucosal	Atezolizumab + Bevacizumab	NCT04091217	*n* = 43	ORR	Phase 2, single-arm
Mucosal	Camrelizumab + Anlotinib + Nab-paclitaxel	NCT04979585	*n* = 66	ORR	Phase 2, single-arm
Stage IV melanoma	Bevacizumab + Nab-paclitaxel vs. Ipilimumab	NCT02158520	*n* = 24	PFS	Phase 2, randomized
Mucosal, cutaneous	Aldesleukin + Pembrolizumab	NCT02748564	*n* = 10	BORR	Phase 2
Mucosal, cutaneous	Carboplatin + Paclitaxel + Sorafenib vs. Carboplatin + Paclitaxel	NCT00110019	*n* = 823	OS	Phase 2, randomized
Mucosal, cutaneous	Ipilimumab + Nivolumab vs. Ipilimumab	NCT03033576	*n* = 94	PFS	Phase 2, randomized
Mucosal, cutaneous, uveal	Epacadostat + Peptide vaccine	NCT01961115	*n* = 11	the number of tumor-infiltrating lymphocytes	Phase 2, single-arm
Mucosal, cutaneous, uveal	APG-115 + Pembrolizumab	NCT03611868	*n* = 224	MTD, RP2D, OS	Phase 1/2
Mucosal, cutaneous	Nivolumab + Ipilimumab + Encorafenib + Binimetinib vs. Nivolumab + Ipilimumab	NCT03235245	*n* = 271	PFS	Phase 2, randomized
Mucosal, acral, cutaneous	Nivolumab + Encorafenib + Binimetinib vs. Nivolumab + Ipilimumab	NCT04511013	*n* = 112	PFS	Phase 2, randomized
Non-ocular melanoma	Tebentafusp + Pembrolizumab vs. Tebentafusp	NCT05549297	*n* = 460	OS, ctDNA reduction	Phase 2, randomized
Mucosal, cutaneous	Albumin-paclitaxel + Ipilimumab	NCT01827111	*n* = 21	mOS, mPFS	Phase 2, single-arm
Non-ocular melanoma	Lifileucel + Pembrolizumab vs. Lifileucel	NCT05398640	*n* = 670	ORR, PFS	Phase 2, randomized
Mucosal, acral	Camrelizumab + Famitinib	NCT05051865	*n* = 60	ORR	Phase 2

*ORR* objective response rate, *PFS* progression-free survival, *OS* overall survival, *BOR* best objective response, *AEs* adverse events, *RP2D* recommended phase 2 dose, *BORR* best overall response rate, *MTD* maximum tolerated dose, *DEC-C* decitabine/cedazuridine

#### Combination of Chemotherapy with Anti-angiogenic Therapy

The combination of anti-angiogenic therapy and chemotherapy has demonstrated synergistic antitumor activity in patients with previously untreated advanced MM. Compared to dacarbazine alone, endostar combined with dacarbazine reduced the risk of death in patients with MM by 93% (HR = 0.07;95% CI = 0.009–0.63) [46]. Moreover, a phase Ⅱ clinical study evaluated the efficacy of bevacizumab combined with paclitaxel plus carboplatin as first-line treatment for advanced MM. Compared with chemotherapy alone, combination with bevacizumab significantly improved clinical outcomes, increasing the ORR from 13.2% to 19.7%, median PFS from 3.0 to 4.8 months, and median OS from 9.0 to 13.6 months [47].

Anti-angiogenic therapy in combination with chemotherapy may still provide clinical benefit for patients with advanced MM who have experienced disease progression following immunotherapy. In a phase Ⅱ clinical trial, the efficacy of apatinib combined with temozolomide were evaluated in patients with advanced melanoma who had progressed after immunotherapy. The median PFS and OS for patients with MM was 4.8 months and 8.0 months, respectively [48]. Another retrospective analysis revealed that the ORR of endostar combined with chemotherapy (dacarbazine or temozolomide plus cisplatin) in treating patients with advanced MM who have progressed after immunotherapy was 30.0%. The median PFS and median OS were 4.9 months and 15.3 months, respectively. Grade 3 or higher TRAEs were observed in 46.6% of patients with MM [49].

#### Combination of Chemotherapy and Immunotherapy

Temozolomide enhances the antitumor efficacy of ICIs by increasing tumor mutational burden to promote immunogenic neoantigens generation and suppressing regulatory T cells within TME [50, 51]. However, despite significantly improved ORR and median PFS when pembrolizumab is combined with temozolomide compared to either agent alone, clinical remission was not observed in patients with MM [52].

Paclitaxel can induce immunogenic cell death through various mechanisms, such as recruiting and activating lymphocytes, promoting the production of immunopotentiating cytokines, and reducing immunosuppressive cells [53, 54]. In addition, paclitaxel can induce the expression of PD-L1 on the surface of tumor cells [55, 56]. Li JJ et al. reported an ORR of 33.3%, and a median PFS of 4.9 months for the combination of a PD-1 inhibitor with albumin-bound paclitaxel in the treatment of advanced MM [57]. In a clinical study, the combination of the LAG-3 inhibitor DNV3, toripalimab, and nab-paclitaxel as first-line treatment for advanced MM yielded an ORR of 38.5%. The incidence of grade 3/4 TRAEs was 22.2% in the overall study population [58].

#### Combination of Immunotherapy with Anti-angiogenic Therapy

Angiogenic factors such as vascular endothelial growth factor (VEGF) drive immunosuppression in the TME by inducing vascular abnormalities, suppressing antigen presentation and immune effector cells, or augmenting the immune suppressive activity of regulatory T cells [59]. Anti-angiogenic therapy blocks the VEGF/VEGFR signaling pathway and inhibits tumor angiogenesis, thereby normalizing the aberrant tumor vasculature. This process improves drug delivery, alleviates tumor hypoxia, and promotes the infiltration of cytotoxic T lymphocytes, ultimately synergizing with immunotherapy [60].

The combination of ICIs and anti-angiogenic agents significantly enhances antitumor activity. Although combination therapy presents a higher risk of TRAEs than monotherapy, it demonstrated an acceptable safety profile. Recent clinical trials have demonstrated that the combination of a PD-1 inhibitor and a VEGFR inhibitor, used as first-line therapy in patients with advanced MM, is associated with an ORR ranging from 35.5% to 50.0%, a median PFS between 5.1 and 11.9 months, and a median OS of 14.3 to 26.8 months [61–66]. In terms of safety, TRAEs for grade ≥ 3 occurred in 33.3% to 67.0% of patients with MM [62, 63]. A retrospective study of 147 patients with MM treated with axitinib plus a PD-1 inhibitor showed that in the first-line setting, the ORR was 30.0%, median PFS was 7.2 months, and median OS was 13.9 months. Among patients receiving subsequent-line therapy, the corresponding values were 17.5%, 4.1 months, and 8.8 months, respectively [67]. Another phase II study using atezolizumab plus bevacizumab in unresectable/advanced MM reported an ORR of 45.0%, median PFS of 8.2 months, and a 1-year OS rate of 76.0%. TRAEs for grade ≥ 3 occurred in 25.6% of patients [68]. Both PD-1 and PD-L1 inhibitors, when combined with anti-angiogenic agents, demonstrate comparable efficacy in the first-line treatment of advanced MM. Notably, a recent retrospective analysis revealed an ORR of 71.4% with lenvatinib plus PD-1 inhibitor in patients with advanced MM whose disease progressed after prior immunotherapy or targeted therapy [69]. However, this promising finding requires further validation in larger patient cohorts. Additionally, a phase I trial of LBL-007 (an anti-LAG-3 antibody) in combination with toripalimab and axitinib in advanced melanoma demonstrated an ORR of 40.0% in the MM subgroup. 45.5% of patients experienced grade ≥ 3 treatment-emergent adverse events (TEAEs) [70]. This regimen did not demonstrate a significant improvement in efficacy compared to the combination therapies mentioned above. The accumulating clinical evidence from combination therapy studies has spurred growing interest in integrating chemotherapy, immunotherapy, and anti-angiogenic therapy for the treatment of advanced MM. A multicenter, single-arm trial of the combination of camrelizumab, anlotinib, and nab-paclitaxel as first-line treatment in advanced MM is currently ongoing [71].

The combination of PD-1 inhibitor and axitinib as a systemic treatment has demonstrated promising antitumor activity in MM. However, its efficacy appears limited in cases with liver metastases, potentially due to the immunosuppressive tumor microenvironment associated with hepatic lesions [67]. Oncolytic virus therapy, which can enhance antitumor immune responses and reverse immunosuppressive conditions, has shown notable efficacy against liver metastases when administered via intrahepatic injection. A phase I study evaluated the efficacy of axitinib combined with pucotenlimab and the oncolytic virus OH2 in patients with liver metastatic MM. 70% of patients had received prior systemic therapy, including 35% who were resistant to PD-1 inhibitors. The ORR was 26.7%. The median PFS was 4.5 months, and the median OS was not reached. The lesion-level response analysis showed a reduction in 43.8% of injected lesions and 23.1% of non-injected lesions. Grade 3/4 TRAEs occurred in 30% of patients, with diarrhea and oral ulceration being the most common [72]. Furthermore, a phase II clinical trial evaluated the safety of adding either ipilimumab or stereotactic body radiotherapy (SBRT) in patients with advanced MM following progression on axitinib plus nivolumab. Specifically, depending on the progression pattern, patients received either SBRT or ipilimumab in addition to continued nivolumab and axitinib. Those with localized or oligometastatic progression received SBRT, while patients exhibiting progression at previously irradiated sites, or with multifocal or disseminated disease not amenable to SBRT, were administered ipilimumab. No grade ≥ 3 TRAEs were observed in the SBRT triple-therapy group, whereas the ipilimumab triple-therapy group exhibited a 40% incidence of grade ≥ 3 TRAEs [66]. This approach demonstrates a manageable safety profile in selected patients with progressive disease, warranting further investigation to overcome anti-PD-1 resistance in MM.

#### Combination of Immunotherapies

CTLA-4 and PD-1 inhibit the anti-tumor immune response through different mechanisms [73]. The CheckMate 067 trial detailed the 5-year long-term outcomes of first-line treatment for patients with MM. Nivolumab plus ipilimumab, nivolumab monotherapy, and ipilimumab monotherapy yielded ORR of 43%, 30%, and 7%, respectively, with corresponding median PFS of 5.8, 3.0, and 2.6 months, median OS of 22.7, 20.2, and 12.1 months, and grade 3/4 TRAE rates of 54%, 26%, and 25% [74]. A retrospective study in patients with advanced MM reported that the ORR for nivolumab and ipilimumab monotherapy were 23.3% and 8.3%, respectively, with median PFS of 3.0 and 2.7 months. In contrast, nivolumab plus ipilimumab achieved an ORR of 37.1% and a median PFS of 5.9 months. The incidence of grade 3/4 TRAEs and treatment discontinuation rate were 8.1% and 2.3% with nivolumab monotherapy, compared to 40.0% and 17.1% with the combination therapy [75]. Despite demonstrating superior efficacy over monotherapy, combination therapy also resulted in a significantly higher incidence of TRAEs.

#### Combination of Immunotherapy with Radiotherapy

Radiotherapy potentiates systemic activation of CD8^+^ T cells by inducing immunogenic cell death and releasing tumor antigens [76]. Furthermore, it upregulates the expression of major histocompatibility complex class I molecules and PD-L1, thereby synergizing with immune checkpoint blockade to significantly enhance disease control of local metastatic lesions [77]. Combination of pembrolizumab and radiotherapy yields a significantly higher target lesion control rate compared to either monotherapy [78]. A retrospective study demonstrated that first-line treatment with a PD-1 inhibitor combined with radiotherapy in patients with advanced MM was associated with an ORR of 27%, a median PFS of 6.8 months, and a median OS of 23.1 months. The rates of grade ≥ 3 TRAEs between the anti-PD1 alone and anti-PD1 plus radiotherapy groups were similar (22% vs. 18%) [24]. In the PORTER-M3 trial, first-line treatment with nivolumab plus radiotherapy for advanced MM resulted in an ORR of 43.8%, a median PFS of 4.9 months, and a median OS of 20.1 months. TRAEs of grades 3/4 occurred in 35.2% of the patients [79]. Combination of PD-1 inhibitors and radiotherapy showed promising efficacy with a manageable safety profile for patients with metastatic MM, and warrants further evaluation in large studies.

## Future Directions

Owing to the unique molecular genetics and an immunosuppressive TME of MM, the response of advanced MM to existing targeted drugs and ICIs is poor. Thus, there is an urgent need to investigate novel treatment strategies. Numerous clinical trials evaluating novel drugs for advanced MM are ongoing (see Table 2).

**Table 2:** Current clinical trials investigating novel agents for unresectable or metastatic mucosal melanoma

Subtype	Agent(s)	NCT number	Sample size	Primary endpoint (s)	Type of study
Mucosal, acral	MGD013	NCT04653038	*n* = 92	ORR	Phase 1, non-randomized
Mucosal, CSD	Nemvaleukin alfa	NCT04830124	*n* = 176	ORR	Phase 2, non-randomized
Mucosal, cutaneous, uveal	VSV-IFNbeta-TYRP1	NCT03865212	*n* = 12	MTD, AEs	Phase 2, non-randomized
Mucosal, cutaneous	TH-302	NCT01864538	*n* = 11	OS	Phase 2
Mucosal, cutaneous, uveal	Olaparib	NCT05482074	*n* = 15	ORR	Phase 2
Mucosal,acral	PLX3397	NCT02071940	*n* = 7	the proportion of tumour progression free at 6 months	Phase 2
Mucosal, acral, cutaneous	Dinaciclib	NCT00937937	*n* = 72	OS	Phase 2
Solid tumors (include MM)	ST101	NCT04478279	*n* = 162	DLT, AEs	Phase 1/2, non-randomized
Mucosal, cutaneous	BO-112 + Pembrolizumab	NCT04570332	*n* = 42	ORR	Phase 2
Mucosal, acral, cutaneous	Lifileucel	NCT02360579	*n* = 178	ORR	Phase 2, non-randomized
Mucosal, acral, cutaneous	Durvalumab + Ceralasertib	NCT03780608	*n* = 30	ORR	Phase 2, non-randomized
Solid tumors (include MM)	Paclitaxel + Ceralasertib	NCT02630199	*n* = 65	MTD, AEs	Phase 1

*ORR* objective response rate, *PFS* progression-free survival, *SC* subcutaneous, *IV* intravenous, *VSV* V esicular Stomatitis Virus, *IFN* interferon, *TYRP-1* tyrosinase related protein 1, *AEs* adverse events, *MTD* maximum tolerated dose, *DLT* dose-limiting toxicity

### Novel Immunotherapy

#### Viral Mimetic

Poly I:C is a double-stranded viral RNA analogue that acts as an agonist of innate immune receptors. BO-112 is a nanocomposite preparation formed by the complexation of Poly I:C and polyethyleneimine. It can enhance the immune response mediated by CD8+ T lymphocytes and systemic cytotoxic T lymphocytes when released in the tumor, thereby inducing tumor cell apoptosis [80, 81]. The ORR of intratumoral injection of BO-112 combined with pembrolizumab in the treatment of patients with advanced MM was 66%. In the overall population, grade 3–5 TRAEs occurred in 9.5% of patients [82].This regimen represents a promising therapeutic strategy for patients with advanced MM who have progressed on prior ICIs.

#### Novel Cytokines

Nemvaleukin alfa is a novel engineered cytokine that selectively binds to a medium-affinity interleukin-2 (IL-2) receptor complex, preferentially activating CD8+ T cells and NK cells while reducing the proliferation of regulatory T cells [83]. It has been approved by FDA as an orphan drug for the treatment of MM. The ARTISTRY-1 study demonstrated anti-tumor activity of nemvaleukin alfa in patients with advanced melanoma, who had previously received ICI treatment. In MM cohorts, the ORR was 17.0%, with a DCR of 66.7% and a median PFS of 13.1 weeks. Despite grade 3–5 TRAEs occurred in 77.0% of patients with melanoma, nemvaleukin alfa had a manageable safety profile with a low incidence of treatment discontinuation (2.0%) due to TRAEs and no adverse events of capillary leak syndrome [84]. These findings support nemvaleukin alfa as a promising single-agent therapeutic option for ICI-resistant patients. Furthermore, the antitumor efficacy of its combination with ICIs in advanced MM warrants further clinical evaluation.

#### Bispecific Antibody

Recently, combination therapy targeting multiple immune checkpoints has been explored to increase anti-tumor efficacy. Particularly, a single molecule targeting more than one checkpoints has been investigated [85]. Preliminary efficacy data from a Phase I study of tebotelimab, a PD-1/LAG-3 bispecific antibody, in patients with previously untreated advanced MM have been reported. The ORR was 30.0%, and the DCR was 50.0%. Grade ≥ 3 and serious TRAEs occurred in 12% and 16% of patients, respectively [86]. Tebotelimab exhibited promising preliminary antitumor activity along with a tolerable safety profile in patients with MM. Moreover, Efficacy results from PD-1/IL-2 bispecific antibody fusion protein IBI363 in patients with advanced melanoma was announced. The overall ORR was 28.1% and DCR was 71.9% [87]. In patients had prior immunotherapy, ORR was 21.2% and DCR was 67.3%. The efficacy data of MM group has not yet been published. Bispecific antibody demonstrated promising antitumor activity in patients with metastatic MM.

#### Tumor-infiltrating Lymphocyte (TIL) Cell Therapy

TIL therapy involves isolating TIL from resected tumors, amplifying these cells using IL-2, and re-infusing them into lympho-depleted patients with IL-2 treatment. Lifileucel, an autologous TIL cell therapy, uses a centralized manufacturing process to produce polyclonal patient-specific TILs derived from TILs extracted from the patients' tumor tissues [88, 89]. The C-144–01 study showed that lifileucel achieved an ORR of 50%, median PFS of not reached and median OS of 19.4 months in patients with advanced MM who had progressed after anti-PD-1/PD-L1 therapy. Hematologic toxicities of grade 3/4 severity were observed in all patients, with recovery to grade ≤ 2 by day 30 following lifileucel infusion occurring in the majority [90]. These data further support the potential benefit of lifileucel as a one-time treatment that is differentiated from other immunotherapies.

#### Chimeric Antigen Receptor (CAR)-T Cell Therapy

CAR can bind to antigens on tumor cells, triggering T cell activation [91]. GD2, a sialic acid-containing ganglioside, is overexpressed in a variety of solid tumor cells, including melanoma [92]. Previous studies have used GD2 as a targeted tumor antigen for CAR-T therapy in melanoma [93]. Yu J et al. found that GD2 expression in MM reached 56.3%, significantly higher than in other melanoma subtypes. Multivariate analysis revealed GD2 as an independent prognostic factor for OS in patients with melanoma. The median OS of patients with ganglioside GD2 expression was significantly shorter compared to those without GD2 expression [31 months vs. 47.1 months) [94]. This study provided a theoretical basis for the treatment of advanced MM with ganglioside GD2 CAR-T cells.

#### Emerging Immunotherapy Targets

Due to the immunosuppressive TME, PD-1/PD-L1 inhibitors alone cannot achieve satisfactory results in patients with advanced MM. The potential value of various novel co-stimulatory receptors and immune inhibitory molecules in the treatment of melanoma has been confirmed [95–113].Drugs targeting these emerging targets, especially in combination with PD-1/PD-L1 inhibitors, may hold promise for MM therapy. Clinical trials of related drugs, both individually and in combination with ICIs, are currently underway (see Supplementary Table 1). It is worth noting that there is no large sample study to detect the expression levels of these immune checkpoints specifically in MM, necessitating further verification of their role in MM treatment.

In recent years, single-cell RNA sequencing has been employed to elucidate the heterogeneity of the immune microenvironment across various subtypes of melanoma. Compared with CM, the infiltration of C-X-C motif chemokine ligand 3-positive tumor-associated macrophages (CXCL3⁺ TAMs) and immunosuppressive neutrophils was significantly increased in MM. These CXCL3⁺ TAMs produced multiple soluble factors, including CXCL3 and transforming growth factor-β (TGF-β) [114]. CXCL3 promotes neutrophil recruitment via binding to C-X-C Motif Chemokine Receptor 2 (CXCR2) [115]. TGF-β can promote the polarization of tumor-associated neutrophils (TANs) recruited by CXCL3 into a pro-tumoral N2 phenotype. TAN-N2 directly inhibits the function of cytotoxic T lymphocytes and natural killer cells by secreting reactive oxygen species, arginase I and a variety of immunosuppressive factors, thereby promoting immune escape [115, 116]. Consequently, targeting the CXCL3–CXCR2 axis along with TGF-β signaling may represent a promising therapeutic strategy for advanced MM. A Phase I trial of the CXCR2 inhibitor SX-682 combined with pembrolizumab for metastatic melanoma (including MM) is ongoing (NCT03161431).

### Novel Targeted Therapy

#### DNA Damage Repair after Failure of Immunotherapy

Tumor cells usually have defects in DNA damage response (DDR). Mutations in DDR genes can lead to increased PD-L1 expression, augmented tumor-infiltrating lymphocytes [117], elevated tumor mutation burden, and enhanced immunogenicity through increased neoantigen burden [118], all of which are potential factors influencing the response to ICIs.

Ataxia Telangiectasia mutated (ATM) and ATR serine/threonine kinase (ATR) are key to the DDR pathway [119], and their inhibitors hold promise for the treatment of advanced MM. In a phase Ⅱ clinical trial, the efficacy of durvalumab combined with the ATR inhibitor ceralasertib were evaluated in patients with advanced melanoma following PD-1 inhibitor treatment failure. The ORR among patients with MM was 40%. The treatment regimen demonstrated a manageable safety profile in the entire study cohort. Grade ≥ 3 TEAEs occurred in 53.3% of patients, all of which resolved with dose interruption and supportive care. No patients discontinued treatment due to TEAEs [120]. In another phase I study, ceralasertib combined with paclitaxel was investigated in patients with advanced melanoma resistant to PD-1 inhibitors. Among patients with MM, the ORR was 45.5% [121].

Poly-ADP-ribose polymerase (PARP) is a core DNA damage sensor in DDR, which binds to damaged DNA lesions, and recruits DNA repair-related protein complex [122]. PARP inhibition not only promotes the accumulation of neoantigens that elicit anti-tumor immunity, but also upregulates interferon signaling, thereby priming the tumor microenvironment to enhance antitumor immune responses [123]. interferons have been shown to induce the expression of PD-L1 [124–126], suggesting a synergistic mechanism between PARP inhibition and immune checkpoint blockade. Furthermore, the upregulation of PARP expression has been significantly associated with decreased survival rates in MM [127]. A phase II clinical trial of the PARP inhibitor Olaparib for the treatment of advanced melanoma (including MM) is currently underway (NCT05482074) and may hold promise for the treatment of advanced MM in the future.

#### Targeting MDM2-p53 Pathway

Inactivation of p53 leads to the abrogation of its tumor-suppressive functions, thereby promoting tumorigenesis and disease progression [128]. Although TP53 mutations are uncommon in MM, the function of p53 is frequently compromised through overexpression of its key negative regulator, mouse double minute 2 homolog (MDM2) [128, 129]. MDM2 amplification, which was observed in 50% of MM samples, may contribute to the poorer responses to ICIs in late-stage MM [129, 130]. As a selective MDM2 inhibitor, alrizomadlin restores p53 function by disrupting the MDM2–p53 interaction, thereby inducing p53-mediated apoptosis in tumor cells harboring wild-type TP53 and/or MDM2 amplification [131]. Alrizomadlin has also been shown to restore anti-tumor activity in patients who have failed PD-1/PD-L1 blockade [132]. Preliminary results from a phase II trial demonstrated an ORR of 40% with alrizomadlin in combination with pembrolizumab in patients with advanced MM who had progressed on prior immunotherapy. The treatment regimen was well tolerated in the overall population. Grade ≥ 3 TRAEs comprised thrombocytopenia (20.2%), neutropenia (14.2%), and anemia (8.3%), with treatment discontinuation due to TRAEs occurring in 5.9% of patients [133]. Collectively, these findings indicate that MDM2 represents a potential therapeutic target in MM.

## Conclusion

MM is characterized by a distinct genomic landscape and a immunosuppressive TME, which contribute to its limited response to existing targeted therapies and ICIs. The combination of PD-1 inhibitors with anti-angiogenic agents or cytotoxic chemotherapy improves outcomes in patients with advanced MM. Several emerging therapeutic strategies show considerable promise in oncology, including viral mimetics, DDR pathway inhibition, MDM2-p53 axis targeting, bispecific antibodies, and TIL therapy. Multiple novel immunotherapeutic targets are under clinical evaluation to address unmet needs in MM treatment. Single-cell sequencing provides a powerful tool for in-depth dissection of the tumor immune microenvironment, facilitating the development of subtype-specific therapies and rational combination strategies to enhance patient survival.

## Key References

- Wei X, Zou Z, Zhang W, et al. A phase II study of efficacy and safety of the MEK inhibitor tunlametinib in patients with advanced NRAS-mutant melanoma. Eur J Cancer. 2024;202:114008.
  - ○ This reference is of importance because it first indicates that tunlametinib demonstrated promising antitumor efficacy and a manageable safety profile in patients with advanced NRAS-mutant mucosal melanoma, providing the rationale for a subsequent randomized controlled trial.
- Lin J et al. Safety and efficacy analysis of DNV3 plus toripalimab and chemotherapy in advanced melanoma: An open-label investigator-initiated trial. J Clin Oncol. 2025;43(16):9529.
  - ○ This reference is of importance because this study provides initial evidence demonstrating the tolerable profile and potential efficacy of combining DNV3 with toripalimab and chemotherapy in patients with advanced melanoma, particularly in subgroups with mucosal melanoma or liver metastases.
- Wang X et al. Axitinib in combination with anti–PD-1 ab (pucotenlimab) plus intra-hepatic injection of oncolytic virus (OH2), in patients with mucosal melanoma and liver metastasis: An open-label phase I trial. J Clin Oncol. 2024;42(16):e21524.
  - ○ This reference is of outstanding importance because this study represents the first clinical trial for liver metastatic mucosal melanoma, aiming to improve the efficacy of combination of anti-PD-1 plus axitinib in patients with liver metastatic mucosal melanoma through intrahepatic injection of oncolytic virus.
- Kluger H, Grigoleit GU, Thomas S, et al. Lifileucel tumor-infiltrating lymphocyte cell therapy in patients with unresectable or metastatic mucosal melanoma after disease progression on immune checkpoint inhibitors. Cancer Commun (Lond). Published online July 22, 2025.
  - ○ This reference is of importance because it shows that lifileucel produces clinically meaningful and durable responses in patients with difficult-to-treat mucosal melanoma who have progressed after anti–PD-1/PD-L1 therapy, supporting its value as a one-time treatment.
- Kim R, Kwon M, An M, et al. Phase II study of ceralasertib (AZD6738) in combination with durvalumab in patients with advanced/metastatic melanoma who have failed prior anti-PD-1 therapy. Ann Oncol. 2022;33(2):193–203.
  - ○ This reference is of importance because it demonstrates that ceralasertib in combination with durvalumab has promising antitumor activity among patients with metastatic mucosal melanoma who have failed anti-PD-1 therapy, suggesting that modulating the DNA damage response and repair pathways is a promising strategy for boosting cancer immunotherapy.
- Li Y, Cui Z, Song X, et al. Single-Cell Transcriptomic Landscape Deciphers Intratumoral Heterogeneity and Subtypes of Acral and Mucosal Melanomas. Clin Cancer Res. 2025;31(12):2495–2514.
  - ○ This reference is of outstanding importance because it identify the specific intratumoral and microenvironmental heterogeneity of acral melanoma and mucosal melanoma, which has profound implications for guiding the development of subtype-specific protocols to improve mucosal melanoma survival.

## Supplementary Information

Below is the link to the electronic supplementary material.

Supplementary file1 (DOCX 47 KB)

## Data Availability

No datasets were generated or analysed during the current study.
