# Supplementary material for: Advanced Mucosal Melanoma Therapies: Current Status and Future Directions
Source: Curr Treat Options Oncol. 2026 Apr 7;27(1):20. doi: 10.1007/s11864-025-01372-y (PMC13056740; doi:10.1007/s11864-025-01372-y)
Supplement: Supplementary file 1 — Supplementary file1 (DOCX 47 KB) [file 11864_2025_1372_MOESM1_ESM.docx]

**Supplementary Table 1. “Co-stimulatory receptors and immune inhibitory molecules that may be used for melanoma treatment.”**

| **Receptor** | **Ligands** | **The role of anti-tumor immune response** | **Ref.** | **Clinical trials** | **Phase** | **Agent** | **Primary Endpoint** |
| --- | --- | --- | --- | --- | --- | --- | --- |
| **Co-stimulatory receptors** |  |  |  |  |  |  |  |
| **GITR** | GITRL | 1. promoting the differentiation and expansion of T cells  2. reducing Treg-mediated immunosuppression | [95] | NCT01239134  NCT03277352  NCT03126110  NCT02697591  NCT03799003 | I  I/II  I/II  I/II  I | TRX518  INCAGN01876  INCAGN01876  INCAGN01876  ASP1951 | AEs  ORR, AEs  ORR, AEs  AEs  AEs, DLT |
| **ICOS** | B7RP-1 | 1. promoting the expansion of effector T cells  2. reducing Treg-mediated immunosuppression | [95] | NCT05695898  NCT03829501  NCT03693612  NCT04319224 | I  I/II  I/II  I/II | XmAb23104  KY1044  Feladilimab  Vopratelimab | AEs, DLT  ORR, AEs, DLT  OS, AEs, DLT  AEs |
| **OX40** | OX40L | 1. stimulating effector T cells  2. increasing the level of multiple cytokines  3. inhibiting immunosuppression of Tregs  4. promoting the cytolytic function of NK cells  5. enhancing NK-mediated antitumor response | [96] | NCT03336606  NCT05661955  NCT03739931  NCT04198766  NCT03894618  NCT04714983  NCT05229601  NCT02315066  NCT02554812  NCT02923349  NCT03241173  NCT02528357 | I  I/II  I  I  I  I  I  I  I/II  I/II  I/II  I | MEDI0562  BGB-A445  mRNA-2752  INBRX-106  SL-279252  DNX-2440  HFB301001  PF-04518600  PF-04518600  INCAGN01949  INCAGN01949  GSK3174998 | activation of immune response  ORR  ORR, AEs, DLT  AEs, DLT, MDT  AEs, DLT, MDT  MDT  AEs, DLT, RP2D  AEs, DLT  ORR, DLT  AEs  ORR, AEs  AEs, DLT |
| **4-1BB** | 4-1BBL | 1. promoting CD8^+^ T-cell proliferation  2. enhancing TCR signaling  3. inducing immunologic memory | [97,98] | NCT00612664  NCT02652455  NCT03809624  NCT01307267  NCT02554812  NCT02315066  NCT04121676 | II  I  II  I  I/II  I  I | BMS-663513  BMS-663513  INBRX-105  PF-05082566  PF-05082566  PF-05082566  AGEN2373 | PFS  AEs  AEs, MDT, RP2D  AEs, DLT  ORR, DLT  AEs, DLT  AEs, DLT |
| **CD40** | CD40L | 1. increasing the tumoricidal activity of macrophages  2. stimulating maturation of APCs | [99] | NCT01103635  NCT03123783  NCT03502330  NCT02706353  NCT04337931  NCT02482168  NCT02376699  NCT03329950 | I  I/II  I  I/II  II  I  I  I | CP-870,893  Sotigalimab  Sotigalimab  Sotigalimab  Sotigalimab  Sotigalimab  SEA-CD40  CDX-1140 | ORR, AEs  ORR, MDT, DLT  AEs  ORR, MDT, RP2D  ORR  AEs, DLT  ORR, AEs  AEs |
| **CD27** | CD70 | 1. promoting the differentiation and expansion of T cells  2. promoting CD8^+^ T cell cytotoxicity | [100] | NCT01460134  NCT02413827  NCT03617328 | I  I/II  I/II | Varlilumab  Varlilumab  Varlilumab | AEs  ORR, AEs  persistent CD4^+^ T cell responses, AEs |
| **immune inhibitory molecules** |  |  |  |  |  |  |  |
| **TIGIT** | CD155  CD112 | 1. triggering T/NK cell-intrinsic inhibition  2. inducing immunosuppressive DCs  3. inhibiting CD226 signaling  4. enhancing immunosuppression of Tregs  5. promoting bacterial Fap2 protein-induced inhibition of T/NK cells | [101,102] | NCT05130177  NCT05665595  NCT04305054  NCT04305041  NCT05483400  NCT04303169  NCT05060432  NCT05116202  NCT05060003  NCT03554083 | II  III  II  II  II  II  II  II  II  II | Domvanalima Vibostolimab  Vibostolimab  Vibostolimab  Tiragolumab  vibostolimab  EOS-448  Tiragolumab  Tiragolumab  Tiragolumab | ORR  RFS  ORR, AEs  ORR, AEs  pCR, ORR  pCR, AEs  ORR, AEs  pRR, ORR  ctDNA clearance rate  pCR, RFS |
| **ADORA2** | Adenosine | 1. inhibiting CD4/CD8 effector T cells and NK cells  2. inhibiting TCR and IL-2 receptor triggered signal  3. enhancing Treg suppressive function  4. inhibiting macrophage antibody-mediated ADCP | [103,104] | NCT03207867  NCT03629756  NCT05955105  NCT05060432 | II  I  I/II  I/II | NIR178  Etrumadenant  ILB2109  Inupadenant | ORR  DLT, AEs  DLT, MDT, RP2D, ORR  DLT, AEs, ORR |
| **VISTA** | VSIG-3  PSGL-1 | 1. enhancing immunosuppression of Tregs  2. inhibiting MHC expression on dendritic cells  3. promoting PD-L1 expression in tumor-associated macrophages  4. inhibiting CD4^+^/CD8^+^ effector T cells  5. downregulating the level of multiple cytokines | [105,106] | NCT02812875  NCT05708950  NCT05864144 | I  I/II  I/II | CA-170  KVA12123  SNS-101 | DLT, MDT, RP2D  AEs, MDT, RP2D  MDT, DLT, AEs, ORR |
| **TIM-3** | Galectin-9  CEACAM  PtdSer  HMGB1 | 1. inhibiting Th1 responses and the expression of cytokines  2. inhibiting NK cells, DCs, and macrophages  3. enhancing Treg suppressive function | [107,108] | NCT04139902  NCT04370704  NCT05451407  NCT03708328  NCT03652077  NCT02608268  NCT02817633 | II  I/II  I  I  I  I/II  I | Cobolimab  INCAGN02390  TQB2618  Lomvastomig  INCAGN02390  Sabatolimab  Cobolimab | MPR  ORR, AEs, DOR, DCR, PFS, ORR, DLT, RP2D  ORR, AEs, DOR, DCR, PFS  AEs, MDT  AEs, DLT, ORR  AEs, DLT, ORR |
| **BTLA** | HVEM | suppressing proliferation and cytokine production in CD8^+^ TILs | [109] | NCT04137900  NCT04773951  NCT05789069 | I  I  I | JS004  JS004  HFB200603 | AEs  AEs, MDT, DLT  AEs, MDT, DLT, RP2D |
| **IDO-1** | − | 1. suppressing T-cell immune responses and multiple cytokines  2. promoting Treg-mediated immunosuppression | [110] | NCT01961115  NCT01604889  NCT05280314  NCT02658890  NCT02178722  NCT03329846 | II  I/II  II  I/II  I/II  III | Epacadostat  Epacadostat  IO102-IO103  BMS-986205  Epacadostat  BMS-986205 | CD8^+^ cells infiltrating tumor  OS, AEs  MPR  ORR, AEs, DOR, PFS  ORR, AEs  AEs |
| **LAG-3** | MHC II  LSECtin  FGL1  Galectin-3 | 1. enhancing the immunosuppressive function of Tregs  2. suppressing multiple cytokines  3. accelerating T cell exhaustion and blocking T cell proliferation | [111] | NCT05704933  NCT05629546  NCT04935229  NCT01968109  NCT03978611  NCT02465060  NCT05704647  NCT05428007NCT05418972  NCT03743766  NCT04552223  NCT05077280  NCT03470922  NCT05625399  NCT05002569  NCT03005782  NCT05352672  NCT05608291  NCT04640545  NCT04370704  NCT04140500  NCT05116202  NCT05419388  NCT03849469  NCT05695898  NCT04618393 | I  I  I  I/II  I  II  II  II  II  II  II  II  II/III  III  III  I  II/III  III  I  I/II  I/II  I/II  I/II  I  I/II  I/II | Relatlimab  Relatlimab  Relatlimab  Relatlimab  Relatlimab  Relatlimab  Relatlimab  Relatlimab  Relatlimab  Relatlimab  Relatlimab  Relatlimab  Relatlimab  Relatlimab  Relatlimab  Fianlimab  Fianlimab  Fianlimab  LBL-007  INCAGN02385  RG-6139  RG-6139  RG-6139  Bavunalimab  Bavunalimab  EMB-02 | immune cell population  AEs  ORR, AEs, MDT, OS  ORR, AEs, DCR, DOR  AEs, DLT  ORR  ORR  ORR, AEs  pCR  ORR  ORR  AEs  PFS  Cavgd28, Cminss  RFS  AEs, DLT  ORR, PFS  RFS  AEs, MDT, DLT  ORR, AEs, DCR, DOR, PFS  ORR, AEs, DCR, DOR, PFS, DLT  pRR, ORR  PFS  AEs  AEs, DLT  ORR, AEs, DLT |
| **CD73** | − | 1. blocking the proliferation and the function of T/NK cells  2. promoting Treg proliferation and suppressive activity | [112,113] | NCT04148937  NCT04797468  NCT04572152  NCT05950815  NCT05173792  NCT06166888  NCT04672434  NCT05119998  NCT03954704  NCT06056323  NCT05174585  NCT04322006  NCT05205109  NCT05559541  NCT05689853  NCT02754141  NCT02503774  NCT06081907  NCT06001580 | I  I  I  I  I  I  I  I  I  I  I/II  I/II  I  I  I/II  I/II  I  I  I | LY3475070  HLX23  AK119  PM1015  AK119  AK131  Sym024  IBI325  GS-1423  HB0045  JAB-BX102  TJ004309  ATG-037  AK119  AK119  BMS-986179  Oleclumab  IBI363  BR101 | AEs, DLT  DLT, RP2D  AEs, DLT  AEs, DLT  AEs, DLT  AEs, DLT  AEs, MTD  AEs, DLT  AEs, DLT  ORR, DLT, MDT  ORR, DOR, AEs, DLT  DLT, MDT  AEs, DLT, MDT, RP2D  ORR, AEs, DLT  ORR, AEs, DLT  AEs  AEs, DLT  ORR, AEs  AEs, DLT, MDT, RP2D |

ORR: objective response rate; RFS: recurrence free survival; pCR: pathological complete remission; pRR: pathological response rate; AEs: adverse events; MTD: maximum tolerated dose; DLT: dose-limiting toxicity. MPR: major pathologic response; DOR: duration of response; DCR: disease control rate; PFS: progression-free survival; OS: overall survival; BTLA: B and T-lymphocyte attenuator; ICOS: inducible T-cell costimulator; LAG-3: lymphocyte-associated gene 3; TIGIT: T cell immunoglobulin and ITIM domain; TIM-3: T-cell immunoglobulin and mucin domain-3; VISTA: V-domain immunoglobulin suppressor of T cell activation; Cminss: trough serum concentration at steady state; Cavgd28: time-averaged serum concentration over 28 days after the first dose; APCs: antigen-presenting cells
